# Research on farmers’ willingness to grow high-quality rice based on the TAM-TPB model: evidence from China

**DOI:** 10.3389/fnut.2025.1535720

**Published:** 2025-07-02

**Authors:** Bin Zhang, Yingshu Cai, Zhen Hu, Ning Xie, Jianfu Li

**Affiliations:** ^1^School of Economics and Management, Jiangxi Agricultural University, Nanchang, Jiangxi, China; ^2^School of Humanities and Public Administration, Jiangxi Agricultural University, Nanchang, Jiangxi, China; ^3^Jiangxi Rural Revitalization Strategy Research Institute, Jiangxi Agricultural University, Nanchang, Jiangxi, China

**Keywords:** occupational grain farmers, high-quality rice, technology acceptance model, theory of planned behavior, SEM

## Abstract

**Introduction:**

Professional grain farmers play a key role in China’s food production, and growing high-quality rice is essential to secure their income. Existing studies have analyzed the willingness of farmers to adopt new rice varieties from a number of perspectives, such as farm characteristics and external factors, and have focused on the overall category of farmers. There are few studies to explore how professional grain farmers induce the planting intention of high-quality rice from the level of subjective psychological mechanism.

**Methods:**

Based on the Technology Acceptance Model-Planning Behavior Theoretical Framework (TAM-TPB), we used the survey data of 660 professional grain farmers in Jiangxi Province, China, and used the structural equation model to explore the influencing factors of the willingness of professional grain farmers to plant high-quality rice, and combined with the multi-group analysis method, we further discussed the differences between different groups.

**Results and discussion:**

The results indicate that the willingness of occupational grain farmers to cultivate high-quality rice is primarily positively influenced by perceived ease of use, perceived usefulness, behavioral attitudes, subjective norms, and perceived behavioral control, with perceived behavioral control having the most significant impact, followed by perceived usefulness. Perceived usefulness positively affects the willingness to grow high-quality rice through behavioral attitudes, while perceived ease of use indirectly influences this willingness via both behavioral attitudes and perceived behavioral control. The multi-group analysis reveals that high-endowment occupational grain farmers exhibit stronger effects of behavioral attitudes and perceived behavioral control on their willingness to grow high-quality rice, whereas low-endowment occupational grain farmers are more significantly influenced by subjective norms and perceived ease of use. Based on these findings, relevant authorities should establish effective communication networks with farmers, enhance their training and education on new varieties and technologies, and refine policy design to promote the willingness of farmers to cultivate high-quality rice.

## 1 Introduction

Rice is a crucial staple food in China, providing sustenance for nearly two-thirds of the population. As the leading rice producer globally, China has witnessed minor variations in its rice output, yet the total production has consistently exceeded 200 million tons for the past 13 years. In 2023, China’s rice production reached 207 million tons, thereby securing the fundamental stability of the nation’s staple food supply. While China’s rice production is sufficient to satisfy the food needs of its domestic population, the historical dependence on a growth model characterized by factor expansion has resulted in notable diminishing marginal returns. As a result, the sustainability of food production growth is now at risk (1). Concurrently, as living standards improve and dietary preferences evolve, domestic demand for rice is transitioning from quantity to quality. While China has ample stocks of ordinary rice, the supply of high-quality rice is becoming increasingly tight, necessitating a shift from a production-centric to a quality-centric approach. The price volatility of high-quality rice, coupled with rising agricultural input costs and social services, has dampened farmers’ enthusiasm for cultivating it. Moreover, standardized production is essential for the high-quality rice industry, yet widespread adoption of harvesting norms, processing standards, and efficient cultivation techniques remains limited. The core of high-quality rice production lies in the adoption of new varieties by farmers, presenting new challenges in rice cultivation. Given that farmers are the primary agents in rice production, addressing the issues in the high-quality rice industry requires a focus on farmers’ willingness, particularly among professional grain farmers, to cultivate high-quality rice and their subsequent behavioral choices.

High-quality rice is characterized by its elongated, slender grains, absence of chalkiness, high amylose content, vivid color, and distinctive aroma upon cooking. These rice varieties are soft and non-sticky, enhancing their palatability (2, 3). Favored by farmers, high-quality rice offers advantages such as increased yield, exceptional grain quality, and strong resistance to pests and diseases (4). It has been reported that high-quality rice can increase grain yield, improve farmers’ profitability, meet increasing food consumption requirements, and guarantee food security (5–7). For instance, Wang et al. (8) used the survey data from Chinese farmers to show that high-quality rice cultivation can raise grain yield and quality. To facilitate the broader adoption of high-quality rice, it is crucial to identify the factors that influence farmers’ decision-making processes.

Scholars argue that farmers’ choices related to rice varieties and technology adoption are influenced by both internal and external factors. The internal factors are farmer individual characteristics, like gender, education level, age, ethnicity, and attitude toward risk, all of which play critical roles in influencing their adoption decisions (9–12). For example, Sall et al. (13) showed that farmers’ perceptions of technological attributes significantly influence their adoption of improved rice varieties in the Casamance region of Senegal. Furthermore, Le et al. (14) established that social networks also impact the adoption of new rice varieties, based on village-level panel data from central Vietnam. External factors, including weather conditions, topography, agricultural extension services, access to credit, and market prices, are also critical in determining farmers’ selection of rice varieties (15–20). For instance, Veettil et al. (21) in their application of an ordered Logit model indicated that improved information quality increases the adoption of stress-tolerant rice varieties. Although these studies have examined the internal and external factors influencing farmers’ willingness to adopt rice varieties, few have integrated these perspectives to explore how farmers’ subjective psychology affects their adoption decisions.

In the theoretical frame studying farmers’ adoption of agricultural technologies, the TAM, TPB, or a combination of both have been used by scholars to study the willingness of farmers to adopt technologies. Some research has established that in the TPB, behavioral attitude, perceived behavioral control, and subjective norm have significant positive impacts on farmers’ willingness to adopt organic agriculture, with subjective norm having a notable impact across different farmer characteristics, whereas the effects of behavioral attitude and perceived behavioral control are less consistent (22). Furthermore, these factors may indirectly influence farmers’ intentions through perceived effects (23); perceived usefulness and perceived ease of use are critical determinants of farmers’ adoption intentions (24). Conversely, other research suggests that perceived usefulness significantly affects farmers’ intentions, while the impact of perceived ease of use is less pronounced (25). Additionally, some research also finds that behavioral attitude, perceived behavior control and perceived economic benefit have a significant impact on farmers’ intentions to adopt, while subjective norms are not significant in impacting farmers’ intentions to adopt or not (26). Thus, farmers’ intention analyses that are based on TAM and TPB provide different conclusions since the same factor may have direct or indirect influences on farmers’ intentions, and the significance of these effects remains contentious. Given that both theoretical models are derived from the Theory of Reasoned Action and exhibit certain complementarities, the integrated TAM-TPB model offers greater explanatory power than either model in isolation (27). On one hand, the TAM effectively measures the factors influencing farmers’ willingness to adopt high-quality rice cultivation; on the other hand, the intention to cultivate high-quality rice aligns with the behavioral intention framework of the TPB. Therefore, investigating farmers’ perceptions, attitude, and adoption intention at the level of farmers using the integrated TAM-TPB model can be a promising research direction.

Despite extensive research on the factors influencing farmers’ adoption of rice varieties, several areas remain underexplored. Firstly, while the Theory of Planned Behavior (TPB) examines the formation of behavioral intentions, the Technology Acceptance Model (TAM) measures the factors influencing farmers’ acceptance of new technologies, which is a highly dynamic system. To enhance the accuracy of the analysis, this study integrates the two models, expands the variables horizontally, and examines the detailed relationships between farmers’ acceptance of new rice varieties and the factors influencing behavioral intentions, distinguishing between direct and indirect effects on adoption intentions. Secondly, although the academic community has analyzed farmers’ willingness to adopt new rice varieties from various perspectives, including farm characteristics and external factors, few studies have applied the TAM-TPB model to investigate farmers’ adoption of high-quality rice, exploring how farmers’ subjective psychological mechanisms drive the willingness to grow high-quality rice. Moreover, given the heterogeneity in farmers’ personal and family endowments, the perception of the same technology may vary significantly, leading to divergent adoption intentions. Thirdly, previous research has predominantly focused on the overall category of farmers without further segmentation, overlooking the group of professional grain farmers. At present, professional grain farmers, characterized by their specialized production capabilities and a stable income, have emerged as the primary contributors to grain production, significantly influencing the sustainable growth of this sector (28, 29).

From the perspective of farmers, we selected 660 professional grain farmers in Jiangxi Province, China, as our research subjects. We integrated the Technology Acceptance Model (TAM) from social psychology with the Theory of Planned Behavior (TPB) to construct a TAM-TPB framework. Employing the path analysis method of structural equation modeling, we examined the psychological decision-making process, key influencing factors, and variations among different types of farmers regarding the adoption of high-quality rice varieties at a subjective psychological level.

## 2 Theoretical framework and hypotheses

The Technology Acceptance Model (TAM), originally formulated by Davis and grounded in the Theory of Reasoned Action and the Theory of Planned Behavior, suggests that an individual’s perceived usefulness and perceived ease of use significantly influence their behavioral intention (30). Over the years, TAM has evolved and been expanded by many researchers into its wider applicability. It is not only applied in predicting individuals’ acceptance of information system technology, but it also denotes the explanation of farmers’ adoption of biological inputs and readiness to embrace green pest control technologies (25, 31).

According to the Theory of Planned Behavior, an extension from the Theory of Reasoned Action by Ajzen, individual behavior is an intentional conduct influenced by both internal and external perceptions through a purposeful tendency-a concept known as behavioral intention. This intention is the product of psychological cognition within an individual, comprising personal attitude, subjective norm, and perceived behavioral control. Consequently, attitudes, subjective norms, and perceived behavioral control collectively determine an individual’s behavioral intention (32). Currently, TPB is pivotal in investigating farmers’ behavioral intentions, serving as a foundational framework for analyzing farmers’ willingness to adopt low-carbon agricultural technologies, pesticide use intentions, and participation in wetland management and protection (33–35).

Since their inception, both the Technology Acceptance Model (TAM) and the Theory of Planned Behavior (TPB) have demonstrated significant explanatory and predictive capabilities in understanding individual behaviors and intentions. However, determining which model possesses greater explanatory power remains challenging. Building on the comprehensive analysis of TAM and TPB by Taylor and Todd (36), the C-TAM-TPB model, which integrates both frameworks, has been introduced to enhance their interaction. Although both models are grounded in the Theory of Rational Behavior, they offer distinct perspectives, making them theoretically compatible and potentially synergistic. This suggests that combining these models can provide a more comprehensive understanding of the various factors influencing individual behavioral intentions (37–39). Recent research has utilized the integrated TAM-TPB model to investigate the impact of subjective factors on farmers’ willingness to adopt rice-shrimp farming practices and the influence of digital meteorological information services on rice farmers’ climate adaptation strategies (37–39). Given that the willingness of professional grain farmers to cultivate high-quality rice exemplifies individual decision-making and cognitive behavior, this study employs the integrated TAM and TPB to clarify the willingness of these farmers to adopt new high-quality rice varieties. The research identifies perceived ease of use, perceived usefulness, behavioral attitude, perceived behavioral control, and subjective norms as latent variables. These variables encompass technological characteristics, social influences, and psychological factors, thereby enabling a more nuanced analysis of the behavioral intentions of professional grain farmers.

### 2.1 Perceived ease of use and perceived usefulness

Perceived ease of use implies an individual’s subjective judgment concerning the ease associated with using a particular technology (37). This concept is relevant to the perceived difficulty or simplicity of adopting new high-quality rice varieties among farmers. The use of new technologies usually necessitates the required time and attention that are related to learning and adaptation. The users are much more likely to try the implementation when they perceive that the technology is easy to use. Conversely, they will have a low intention of adoption. Researchers have found that perceived ease of use may reduce farmers’ apprehension about a new technology and enhance their confidence in their ability to use such technologies, leading to an increased likelihood of adoption (40). For professional grain farmers, perceived ease of use in cultivating high-quality rice involves their perception of the difficulty or ease of adopting new techniques and methods for growing high-quality rice varieties. When professional grain farmers believe they can manage the cultivation of high-quality rice without much effort-that is, meet environmental requirements for high-quality rice, implement scientific fertilization and irrigation practices, and adopt more meticulous monitoring and preventive measures-their intention to adopt high-quality rice cultivation will be higher. Based on this literature background, we propose the following hypothesis:

*H1*: Perceived ease of use has a significant positive impact on the willingness of professional grain farmers to cultivate high-quality rice.

Perceived usefulness, as defined academically, refers to the extent to which farmers subjectively perceive that adopting new rice varieties can enhance effectiveness and utility (41). Empirical research has established that perceived usefulness is a crucial determinant significantly influencing individuals’ inclination to adopt novel technologies (42). Unlike conventional technologies, new technologies are only embraced if they offer superior returns to farmers; otherwise, their adoption intention diminishes. Similarly, in line with the rational smallholder theory, perceived usefulness also shapes the willingness of professional grain farmers to cultivate high-quality rice. Ordinary rice, characterized by its inferior pest and disease resistance, necessitates substantial chemical fertilizers and pesticides. Moreover, its taste and nutritional value are subpar compared to high-quality rice, resulting in diminished consumer demand and limited market potential (65). Consequently, the economic returns for farmers cultivating ordinary rice are constrained. In contrast, high-quality rice varieties exhibit robust disease resistance, high yield potential, and strong growth characteristics (38, 39). The cultivation of high-quality rice not only optimizes land and capital utilization but also garners widespread consumer favor due to its superior nutritional value and taste, leading to a burgeoning acreage of high-quality rice in recent years and its gradual ascendancy as the predominant reserve grain variety (38, 39). Consequently, professional grain farmers who opt to plant high-quality rice can curtail production and operational costs, augment yields, and elevate planting returns, thereby fostering a heightened willingness to adopt high-quality rice cultivation. Therefore, this study expects that:

*H2*: Perceived usefulness has a significant positive impact on the willingness of professional grain farmers to cultivate high-quality rice.

### 2.2 Behavioral attitudes, subjective norms, and perceived behavioral control

The Theory of Planned Behavior posits that an individual’s behavioral intention and behavior are outcomes of a deliberative process shaped by behavioral attitudes, subjective norms, and perceived behavioral control (43). Behavioral attitude represents an individual’s predisposition to engage in a specific behavior, which, in the context of professional grain farmers, pertains to their positive or negative evaluation of cultivating high-quality rice (44). Farmers typically weigh various factors when making decisions, including the balance between anticipated benefits and costs (45). When farmers perceive a certain action as advantageous, they develop a positive behavioral attitude, thereby enhancing their readiness to perform that behavior. For example, Zhang et al. (46) developed an enhanced TPB framework, illustrating that the perceived benefits of high-quality agricultural products among large-scale farmers can foster a positive behavioral attitude, which subsequently positively affects their intention to produce high-quality agricultural products. Consequently, this study hypothesizes:

*H3*: Behavioral attitudes significantly and positively influence the willingness of professional grain farmers to cultivate high-quality rice.

Subjective norms encompass the pressures farmers face during the cultivation of high-quality rice, influenced by factors such as conformity, suggestion, and imitation. These norms include prescriptive and descriptive norms (47). Prescriptive norms are the pressures perceived by farmers from authoritative sources, such as government agencies and agricultural extension personnel, advocating for the cultivation of high-quality rice. Given the expertise and authority of agricultural extension personnel, their guidance is crucial in steering farmers toward cultivating high-quality rice. Descriptive norms, on the other hand, arise from the demonstration effects of neighboring farmers and acquaintances who engage in the cultivation of high-quality rice. The positive feedback and successful experiences shared by these individuals significantly boost farmers’ inclination to cultivate high-quality rice. Based on this, we hypothesize:

*H4*: Subjective norms significantly and positively impact the willingness of professional grain farmers to cultivate high-quality rice.

Perceived behavioral control refers to farmers’ subjective assessment of their ability to cultivate high-quality rice, encompassing their perceived mastery over resource endowments, professional skills, and other capital and capability factors. Empirical evidence indicates that perceived behavioral control positively influences farmers’ behavioral intentions. As farmers perceive an increase in their controllable factors, anticipated barriers diminish, thereby enhancing their perceived behavioral control and subsequently increasing the likelihood of behavioral and intention manifestation (48). Certain studies have confirmed, through the lens of the TPB, that perceived behavioral control significantly impacts farmers’ intentions to adopt conservation tillage practices (49). Broadly, the greater the perceived behavioral control of professional grain farmers during the cultivation of high-quality rice, and the more external factors they anticipate being able to manage, the stronger their inclination to cultivate high-quality rice. Therefore, we hypothesize:

*H5*: Perceived behavioral control significantly and positively impacts the willingness of professional grain farmers to cultivate high-quality rice.

### 2.3 Mediation effect analysis

According to the Technology Acceptance Model, perceived usefulness and perceived ease of use are pivotal factors influencing professional grain farmers’ attitudes toward cultivating high-quality rice. Davis (50) highlights that perceived ease of use significantly impacts perceived usefulness. When professional grain farmers have a good mastery of high-quality rice cultivation technology and are well informed about the nature of high-quality rice varieties and their specific environmental requirements, their evaluation of ease of use will be higher, enhancing perceived usefulness and thus attitude toward adopting high-quality rice varieties. Caffaro et al. (51) underline that perceived usefulness is a determinant of farmers’ attitudes. When professional grain farmers think that planting high-quality rice can increase yields and create more value, their perceived usefulness of high-quality rice varieties increases, which further develops a better attitude toward high-quality rice planting. On the contrary, if farmers believe that high-quality rice varieties have no advantages over ordinary rice and do not bring higher perceived benefits, they may develop a negative attitude toward planting high-quality rice varieties. According to the Theory of Planned Behavior, perceived behavioral control reflects an individual’s perception of facilitators or barriers, contingent upon the perceived ease or difficulty of performing a specific behavior (52). When professional grain farmers perceive that cultivating high-quality rice is straightforward to operate, they will also experience a greater sense of control over this behavior, resulting in outcomes more aligned with their expectations and thereby increasing their willingness to adopt high-quality rice varieties. Based on the above analysis, we propose the following hypotheses:

*H6a*: Perceived usefulness influences professional grain farmers' willingness to grow high-quality rice through the mediating role of behavirol attitude.

*H6b*: Perceived ease of use influences professional grain farmers' willingness to grow high-quality rice through the mediating role of behavirol attitude.

*H6c*: Perceived ease of use influences professional grain farmers' willingness to grow high-quality rice through the mediating role of perceived behavioral control.

**Figure 1:**
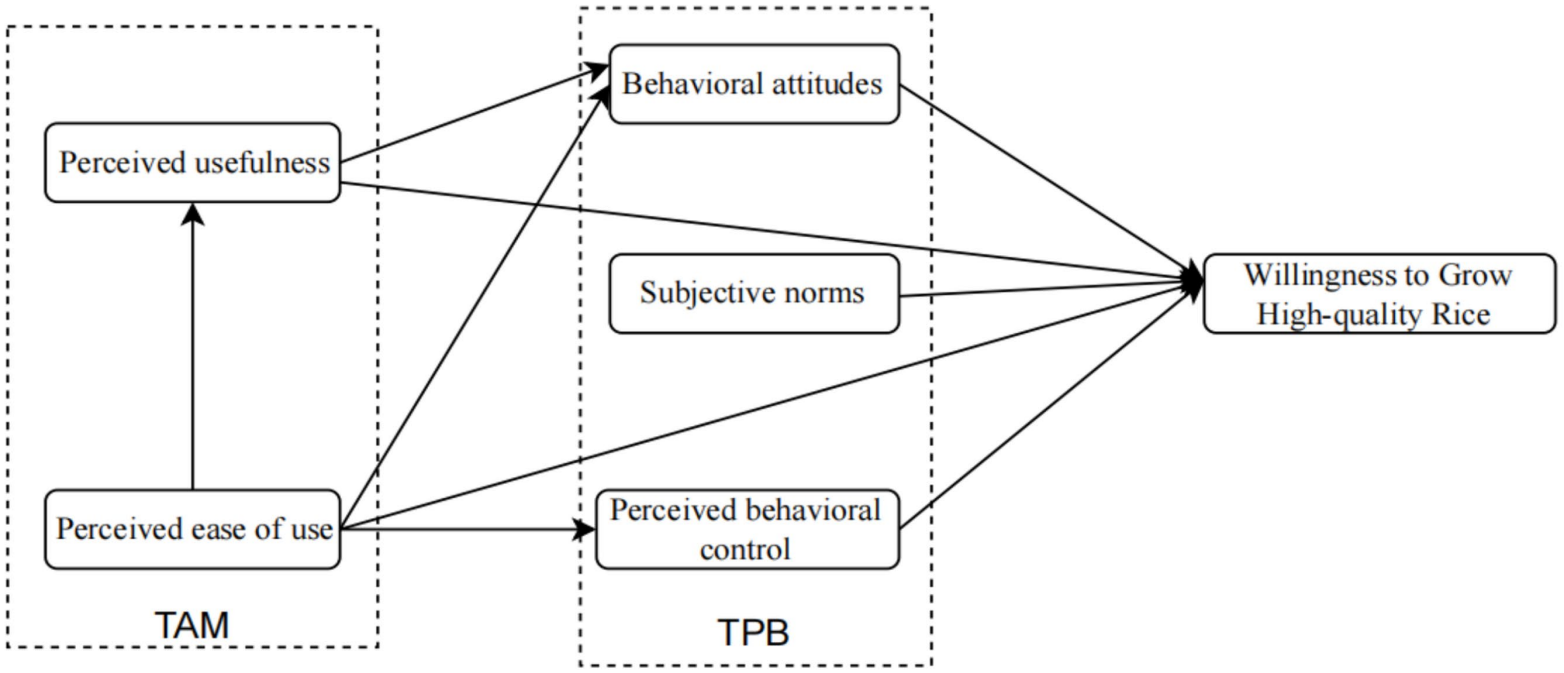
Research framework.

Based on these hypotheses, this study constructs a conceptual model of the factors influencing professional grain farmers’ willingness to grow high-quality rice, grounded in the Technology Acceptance Model and the Theory of Planned Behavior (see Figure 1).

## 3 Materials and methods

### 3.1 Sampling and data collection

**Figure 2:**
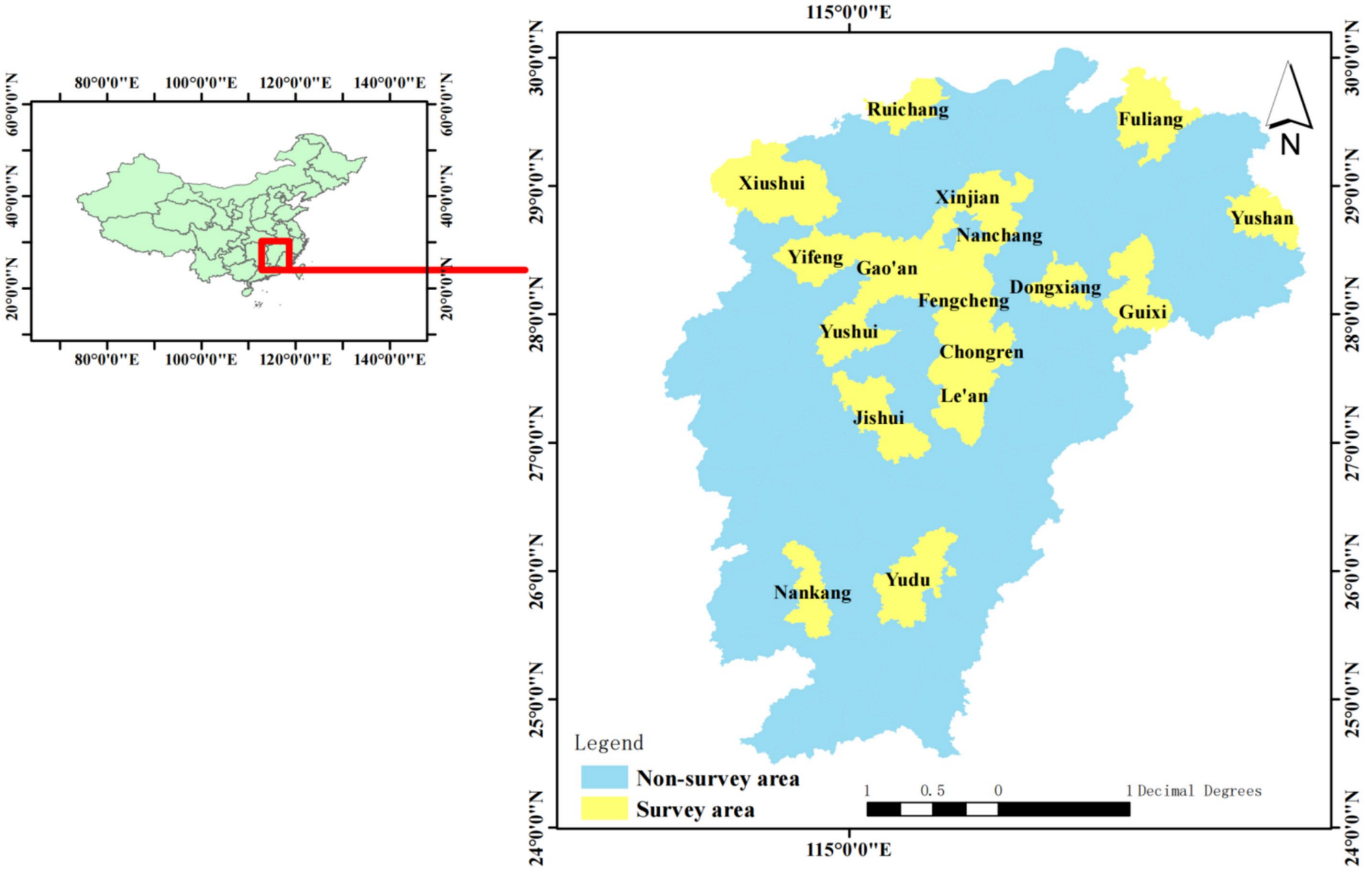
Research area.

This study centered on Jiangxi Province in eastern China, chosen for its designation as one of the country’s 13 major grain-producing regions, ranking third nationally in both rice planting area and total output. Jiangxi’s advantageous natural conditions foster the development of high-quality rice. The Jiangxi provincial government has prioritized agricultural advancement, particularly the cultivation of high-quality rice, and has emphasized the promotion of such rice in recent policy frameworks. Given that high-quality rice represents a novel technological adoption for farmers, it is essential to identify the factors influencing farmers’ decisions to adopt this technology. The survey investigated high-quality rice varieties currently promoted in Jiangxi Province, encompassing early rice varieties such as Xianghe Youmingyue Simao, Xiangzao Xian 45, and Zhongjiazao 17; mid-season varieties including Longliang Youhuazhan and Jingliang Youhuazhan; and late rice varieties like Yexiang Youhang 1,573, Taiyou 398, Taiyou Hang 1,573, and Huanghuazhan. Given that some professional grain farmers struggled to accurately identify whether the varieties they cultivated were high-quality, the research team provided training for surveyors on rice variety identification prior to the survey. Furthermore, the questionnaire was enhanced to include two new elements: “Seed Name” and “Surveyor’s Judgment on Whether It Is a High-Quality Rice Variety.” This study conducted a comprehensive evaluation of the quality of rice varieties by utilizing real data on seed prices and quantities. The research team conducted a preliminary survey to refine the questionnaire before the formal survey, which took place from June to August 2023. Following the methodology outlined by Zhang (29), we selected 4–5 townships from 17 major grain-producing counties in Jiangxi Province, resulting in a total of 77 townships with a planting area exceeding 2 hectares, for interviews and questionnaire surveys (refer to Figure 2). A total of 691 questionnaires were distributed, and after excluding those with missing values, inconsistent responses, or brief completion times, 660 valid questionnaires were obtained, yielding an effective recovery rate of 95.51%.

Among the respondents, 69.1% were male and 30.9% were female, indicating a male-dominated professional grain farmer population. 11.6% of the respondents were under 40 years old, while 88.4% were 41 years old or older, highlighting a significant aging trend among professional grain farmers. A total of 361 farmers, constituting 54.7%, had an education level of primary school or below, reflecting a low educational attainment among professional grain farmers. The cultivated land area of professional grain farmers was predominantly concentrated between 16 and 20 hectares, accounting for 43.3%.

### 3.2 Questionnaires and measurements

All variables in this study were assessed using well-established scales. Following feedback from expert grain farmers during the preliminary survey and subsequent reliability and validity tests, certain measurement items were eliminated. Furthermore, the wording of the items was systematically refined to align with the actual production practices of farmers cultivating high-quality rice. All scales employed a Likert scale, with each item offering five response options: “Very Consistent,” “Consistent,” “Neutral,” “Inconsistent,” and “Very Inconsistent,” coded numerically as 5, 4, 3, 2, and 1, respectively. The measurement items for the variables are detailed in Table 1.

**Table 1:** Questionnaire.

Constructs		Measurement items	Average value	References
Perceived ease of use (PEU)	PEU1	You believe that growing high-quality rice is relatively straightforward.	3.834	Pavlou (61) Chen et al. (62)
	PEU2	Growing high-quality rice does not require excessive effort.	3.905	
	PEU3	You can easily obtain human resources and rice cultivation techniques.	3.627	
Perceived usefulness (PU)	PU1	Growing high-quality rice can increase household income.	3.866	Pavlou (61) Chen et al. (62)
	PU2	Growing high-quality rice can improve the family’s living environment.	4.044	
	PU3	Growing high-quality rice can enhance rice yield.	3.982	
Behavioral attitudes (BA)	BA1	There is a psychological expectation of profit from high-quality rice.	3.849	Xia et al. (22) Senger et al. (63) Tama et al. (49)
	BA2	Growing high-quality rice can improve market competitiveness.	3.553	
	BA3	You believe that growing high-quality rice is highly effective in improving rice quality.	3.724	
Perceived behavirol control (PBC)	PBC1	You feel capable of growing high-quality rice.	3.817	Xia et al. (22) Senger et al. (63) Tama et al. (49)
	PBC2	You can obtain sufficient resources to grow high-quality rice (such as funds, knowledge, etc.).	3.606	
	PBC3	Under your conditions, successfully growing high-quality rice is not difficult.	3.778	
Subjective norms (SN)	SN1	Your family believes you should grow high-quality rice.	3.486	Xia et al. (22) Senger et al. (63) Tama et al. (49)
	SN2	Your fellow farmers generally support growing high-quality rice.	3.496	
	SN3	Agricultural experts or advisors recommend that farmers grow high-quality rice to increase income.	3.417	
Willingness to grow high-quality rice (WGHR)	WGHR1	You are able to actively participate in growing high-quality rice.	3.447	Peng et al. (64)
	WGHR2	You can systematically learn high-quality rice cultivation techniques.	3.583	
	WGHR3	You will grow high-quality rice under the guidance of professionals.	3.287	

### 3.3 Data analysis methods

The structural equation model (SEM) consists of a structural model and a measurement model. The structural model examines the causal relationships among latent variables, which include exogenous latent variables representing “causes” and endogenous latent variables representing “effects” that cannot be directly measured. The measurement model, which typically involves latent variables and observed variables, reflects the relationship between these latent variables and the directly measurable observed variables. This study constructs a structural model to evaluate the causal relationships between exogenous latent variables (perceived ease of use, perceived usefulness, behavioral attitudes, perceived behavioral control, subjective norms) and an endogenous latent variable (willingness to grow high-quality rice), as shown in Equation (1).

(1) η=βη+Γξ+ξ

In Equation (1), *β* represents the coefficient matrix for the relationships between endogenous latent variables, Г represents the coefficient matrix for the relationships between exogenous latent variables *ξ* and endogenous latent variables *η*, and *ξ* represents the measurement error. Since latent variables are difficult to measure directly, multiple observed variables are required. Therefore, measurement models (2) and (3) are established, where *λx* and *λY* denote the relationship matrices between latent variables and observed variables, and δ and ε denote the error terms.

(2) X=λxξ+δ

(3) Y=λYη+ε

The evaluation of convergent validity in structural equation modeling is based on two key indicators: Composite Reliability (CR) and Average Variance Extracted (AVE). According to the standards set by Shi et al. (53), the CR should be at least 0.7, while the AVE should be no less than 0.5. Additionally, this study assesses the model fit using five supplementary indicators computed by AMOS 24.0, with the benchmarks for these indicators detailed in Table 2.

**Table 2:** Benchmarks for model fit assessment indicators.

Model fit assessment indicators	CMIN/DF	RMSEA	NFI	GFI	CFI
Benchmarks	<5	<0.08	>0.8	>0.8	>0.8

## 4 Results

### 4.1 Confirmatory factor analysis

#### 4.1.1 Reliability and validity analysis

The KaiserMeyer-Olkin (KMO) and Bartlett’s Test of Sphericity were performed on the questionnaire items using SPSS20.0 software. The KMO values for the six subscales and the total scale were 0.721, 0.701, 0.653, 0.707, 0.707, 0.904, and 0.733, respectively. The *p*-values obtained from Bartlett’s Test of Sphericity were consistently 0.000. Additionally, the reliability assessment indicated that the Cronbach’s alpha values for each scale item ranged from 0.763 to 0.883. These findings collectively indicate that the questionnaire items are suitable for confirmatory factor analysis. Confirmatory factor analysis was conducted on the measurement model using AMOS24.0 software, and composite reliability (CR) and average variance extracted (AVE) were calculated to assess convergent validity. As presented in Table 3, all measurement indicators in the model met the statistical criteria of 0.5 < factor loading < 0.95, CR > 0.6, and AVE > 0.5, indicating satisfactory convergent validity and an optimal internal quality of the model.

**Table 3:** Reliability and validity analysis.

Constructs	Items	S. Estimate	Estimate	S. E.	C. R.	P	SMC	CR	AVE
Perceived ease of use	PEU1	0.823	1				0.677	0.861	0.675
	PEU2	0.829	0.997	0.04	25.09	***	0.687		
	PEU3	0.812	1.092	0.048	22.739	***	0.659		
Perceived usefulness	PU1	0.776	1				0.602	0.854	0.661
	PU2	0.859	0.962	0.043	22.139	***	0.738		
	PEU3	0.812	1.092	0.048	22.739	***	0.659		
Behavioral attitudes (BA)	BA1	0.729	1				0.531	0.769	0.530
	BA2	0.616	0.895	0.07	12.762	***	0.379		
	BA3	0.824	1.214	0.082	14.762	***	0.679		
Perceived behavirol control (PBC)	PBC1	0.728	1				0.530	0.833	0.626
	PBC2	0.787	1.161	0.061	18.978	***	0.619		
	PBC3	0.853	1.147	0.058	19.74	***	0.728		
Subjective norms (SN)	SN1	0.694	1				0.482	0.850	0.656
	SN2	0.874	1.156	0.061	18.919	***	0.764		
	SN3	0.85	1.184	0.063	18.787	***	0.723		
Willingness to grow high-quality rice (WGHR)	WGHR1	0.878	1				0.771	0.883	0.716
	WGHR2	0.829	0.837	0.032	26.333	***	0.687		
	WGHR3	0.83	0.938	0.035	26.485	***	0.689		

#### 4.1.2 Discriminant validity test

First, confirmatory factor analysis (CFA) was performed using AMOS 24.0 software to evaluate the discriminant validity among perceived ease of use, perceived usefulness, behavioral attitudes, perceived behavioral control, subjective norm, and the intention to plant high-quality rice. The absolute fit statistics of the structural equation model (SEM) were employed to assess the fit between the hypothesized theoretical model and the empirical data. As shown in Table 3, significant correlations were observed among the latent variables (*p* < 1%), with correlation coefficients exhibiting absolute values less than 1%. The square roots of the average variance extracted (AVE) for each variable (highlighted in bold on the diagonal in Table 4) consistently exceeded the correlation coefficients with other variables, indicating that the latent variables are both correlated and distinct. This finding supports the satisfactory discriminant validity of the questionnaire data. Second, correlation analysis among the variables revealed that perceived ease of use was significantly and positively correlated with perceived usefulness, perceived behavioral control, behavioral attitudes, and the intention to plant high-quality rice. Perceived usefulness was significantly and positively correlated with behavioral attitudes and the intention to plant high-quality rice. Furthermore, behavioral attitudes, perceived behavioral control, and subjective norm were significantly and positively correlated with the intention to plant high-quality rice. These results provide preliminary validation for hypotheses H1, H2, H3, H4, and H5, thereby supporting the appropriateness of proceeding with regression model testing.

**Table 4:** Discriminant validity.

Constructs	AVE	PEU	PU	PBC	SN	BA	WGHR
PEU	0.675	**0.822**					
PU	0.661	0.698***	**0.813**				
PBC	0.626	0.732***	0.622	**0.791**			
SN	0.656	0.512	0.357	0.456	**0.810**		
BA	0.53	0.442**	0.511***	0.393	0.226	**0.728**	
WGHR	0.716	0.539*	0.6***	0.555**	0.398***	0.53***	**0.846**

*, **, and *** indicate the significate level of 10, 5, and 1%, respectively. Values on diagonal indicate the square root of the AVE.

#### 4.1.3 Discriminant validity test

Table 5 presents the fit indices for the six-factor model as follows: CMIN/DF = 4.002 (≤ 5), RMSEA = 0.068 (≤ 0.08), NFI = 0.932 (> 0.8), GFI = 0.924 (> 0.8), CFI = 0.948 (> 0.9). These indices collectively indicate an adequate model fit. Additionally, the six-factor model demonstrates the lowest CMIN/DF and RMSEA values, as well as the highest NFI, GFI, and CFI values compared to other models, suggesting superior fit. In contrast, the fit indices for the single-factor model did not meet the statistical criteria (CMIN/DF = 19.373 > 5, RMSEA = 0.167 > 0.08), indicating a poor fit. This finding suggests that the model cannot be adequately explained by a single factor and implies that the common method bias among the six variables in this study is not substantial.

**Table 5:** Common method variance test results.

Model	CMIN	DF	CMIN/DF	RMSEA	NFI	GFI	CFI
Six-Factor Model PEU, PU, BA, PBC, SN, WGHR	481.961	120	4.002	0.068	0.932	0.924	0.948
Five-Factor Model PEU, PU, BA, PBC, SN + WGHR	1150.952	125	9.208	0.112	0.838	0.822	0.852
Four-Factor Model PEU, PU, BA, PBC + SN + WGHR	1749.144	129	13.559	0.138	0.754	0.737	0.767
Three-Factor Model PEU, PU, BA+PBC + SN + WGHR	2058.146	132	15.592	0.149	0.710	0.708	0.723
Two-Factor Model PEU, PU + BA+PBC + SN + WGHR	2329.942	134	17.388	0.158	0.672	0.675	0.684
Single-Factor Model PEU + PU + BA+PBC + SN + WGHR	2615.337	135	19.373	0.167	0.632	0.652	0.643

Model adaptation indicators: CMIN/DF<5, RMSEA<0.08, NFI≥0.8, GFI≥0.8, CFI>0.9.

### 4.2 Structural equation model assessment

**Figure 3:**
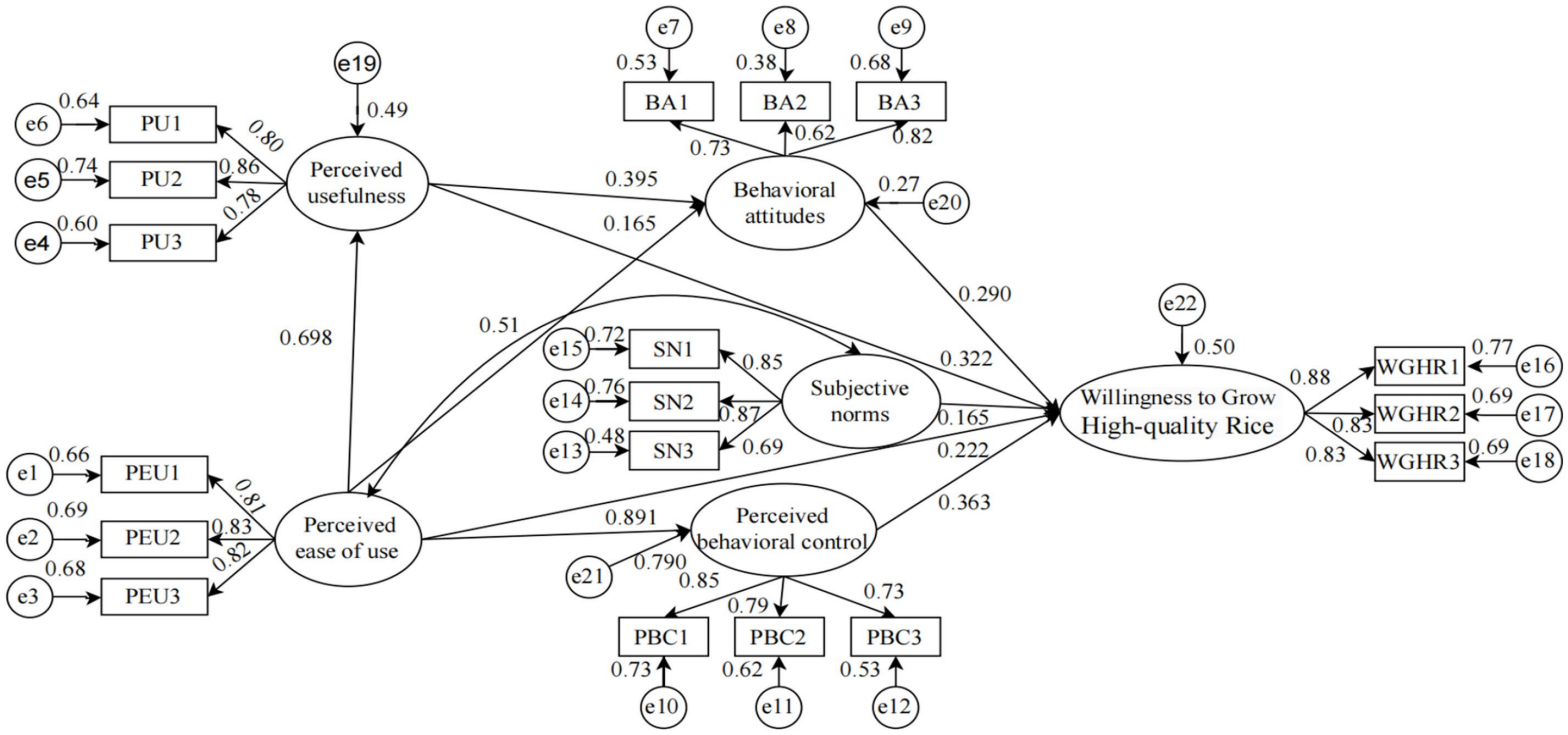
Structural equation model of willingness to grow high-quality rice.

Good model fit is essential for the application of Structural Equation Modeling (SEM) to validate theoretical constructs. In this study, five fit indices were utilized to evaluate the overall model adequacy. The findings indicate that CMIN/DF = 4.237, which is below the threshold of 5; NFI = 0.925, GFI = 0.917, and CFI = 0.942, all of which exceed the benchmark of 0.9; and RMSEA = 0.070, which meets the criterion of being less than 0.08. These indices collectively suggest that the model demonstrates a satisfactory fit, conforming to the requirements of SEM. The empirical validation of the research hypotheses and theoretical framework, conducted using AMOS 24.0 software, is illustrated in Figure 3 and summarized in Table 6.

**Table 6:** Results of the structural model analysis.

Relationship	S. Estimate	Estimate	S. E.	C. R.	Sig.	Result
PEU → PU	0.698	0.868	0.058	15.026	***	Supported
PU → BA	0.395	0.415	0.075	5.51	***	Supported
PEU → PBC	0.891	0.87	0.051	17.163	***	Supported
PEU → BA	0.165	0.216	0.085	2.534	**	Supported
BA→WGHR	0.29	0.332	0.053	6.204	***	Supported
SN → WGHR	0.165	0.222	0.058	3.858	***	Supported
PBC → WGHR	0.363	0.555	0.175	3.166	**	Supported
PEU → WGHR	0.222	0.332	0.187	1.771	*	Supported
PEU → WGHR	0.322	0.387	0.072	5.339	***	Supported

*, **, and *** indicate the significate level of 10, 5, and 1%, respectively.

The empirical results reveal that perceived ease of use significantly positively influences the willingness of professional grain farmers to cultivate high-quality rice (*β* = 0.222, *p* < 0.1), thereby supporting Hypothesis H1; perceived usefulness significantly positively influences the willingness of professional grain farmers to cultivate high-quality rice (*β* = 0.322, *p* < 0.01), supporting Hypothesis H2; behavioral attitudes significantly positively influences the willingness of professional grain farmers to cultivate high-quality rice (*β* = 0.29, *p* < 0.01), supporting Hypothesis H3; subjective norms significantly positively influence the willingness of professional grain farmers to cultivate high-quality rice (*β* = 0.165, *p* < 0.01), supporting Hypothesis H4; and perceived behavioral control significantly positively influences the willingness of professional grain farmers to cultivate high-quality rice (*β* = 0.363, *p* < 0.05), supporting Hypothesis H5.

### 4.3 Analysis of mediating effects

This study utilized the Bootstrap test method to resample 5,000 times within a 90% confidence interval. A significant mediating effect was indicated when the product of coefficients from the Bootstrap samples did not include zero within the 90% confidence interval (54). As presented in Table 7, the mediating effect of the path “prceived usefulness→behavioral attitude→ willingness to grow high-quality rice” was significant, with an effect size of 0.138 and a 90% confidence interval of [0.073, 0.240], thereby supporting Hypothesis H6a. Similarly, the mediating effect of the path “perceived ease of use→behavioral attitude→willingness to grow high-quality rice” was significant, with an effect size of 0.072 and a 90% confidence interval of [0.017, 0.159], supporting Hypothesis H6b. Furthermore, the mediating effect of the path “perceived ease of use→ perceived behavioral control→willingness to grow high-quality rice” was significant, with an effect size of 0.483 and a 90% confidence interval of [0.071, 0.956], supporting Hypothesis H6c.

**Table 7:** Results of the mediation effect test.

Paths	Coefficient	Bias-corrected 90% CI		
		Lower	Upper	*P*
PU → BA→WGHR	0.138	0.073	0.240	0.000
PEU → BA→WGHR	0.072	0.017	0.159	0.031
PEU → PBC → WGHR	0.483	0.071	0.956	0.055
Total effect	1.148	0.668	1.595	0.002

### 4.4 Multiple-group analysis

Research has shown that farmers’ endowments play a crucial role in their acceptance and adoption of agricultural technologies (55). Farmers with differing resource endowments often display distinct behavioral attitudes during the decision-making process. This study adopts the classification approach proposed by Zhang et al. (56) to categorize professional grain farmers based on three indicators of resource endowment: years of education, annual household income, and rice cultivation area. Through multi-group analysis, the research uncovers variations in the willingness to cultivate high-quality rice among professional grain farmers with different resource endowments. Employing the K-means clustering method from hierarchical clustering, professional grain farmers are segmented into two distinct groups: high-endowment and low-endowment. To ascertain whether significant differences exist between these two groups in the aforementioned indicators, the Mann–Whitney U test, a non-parametric statistical test, was utilized. The results of the hypothesis test rejected the null hypothesis, confirming significant disparities in years of education, annual household income, and rice cultivation area between the two groups of professional grain farmers. The clustering outcomes are detailed in Table 8. The first group, characterized by an average education of 4.68 years, an average annual household income of 34,200 yuan, and an average rice cultivation area of 6.52 hectares, is classified as low-endowment professional grain farmers. Conversely, the second group, with an average education of 6.54 years, an average annual household income of 67,000 yuan, and an average rice cultivation area of 10.88 hectares, is classified as high-endowment professional grain farmers.

**Table 8:** Hierarchical clustering results.

Group	Sample Size	Years of education	Annual per capita household income (in 10,000 yuan)	Paddy field area (hectares)
Low-endowment	320	4.68	3.42	6.52
High-endowment	340	6.54	6.7	10.88

Using multi-group analysis in structural equation modeling, we explored the differences in the willingness to cultivate high-quality rice between the two groups of professional grain farmers. The model’s CMIN/DF value is 3.260, RMSEA value is 0.041, and the NFII, GFI, and CFI values are all above 0.9, indicating good model fit.

As shown in Table 9, there are significant differences in the path coefficients between the high-endowment and low-endowment groups. Specifically, the influence coefficients of behavioral attitude and perceived behavioral control on the willingness to grow high-quality rice are significantly greater for high-endowment professional grain farmers compared to low-endowment ones. Conversely, for low-endowment professional grain farmers, the influence coefficients of subjective norms and perceived ease of use on the willingness to grow high-quality rice are stronger than those for high-endowment farmers.

**Table 9:** Results of multi-group analysis.

Paths	High-endowment		Low-endowment	
	Coefficient	*P*	Coefficient	*P*
PEU → PU	0.704	***	0.687	***
PEU → BA	0.377	ns	0.417	ns
PEU → PBC	0.889	***	0.886	**
PEU → BA	0.16	**	0.173	*
BA→WGHR	0.318	***	0.282	**
SN → WGHR	0.378	***	0.565	***
PBC → WGHR	0.380	***	0.315	***
PEU → WGHR	0.156	***	0.221	***
PU → WGHR	0.235	**	0.224	**

*, **, and *** indicate the significate level of 10, 5, and 1%, respectively; ns indicates that the P-value is not significant.

## 5 Discussion

This study integrated the Technology Acceptance Model (TAM) and the Theory of Planned Behavior (TPB) to empirically investigate the willingness of 660 professional grain farmers in Jiangxi Province to cultivate high-quality rice. The research not only validates the applicability of TAM and TPB in the context of agricultural technology adoption but also extends the explanatory scope of these theories in agricultural behavioral decisions. Our findings confirm Davis et al.’s assertions, where perceived ease of use and perceived usefulness of technology are deemed critical determinants of behavioral intentions (30). In the agricultural domain, farmers are bound to adopt technologies they perceive to be friendly and useful. Moreover, behavioral attitudes, subjective norms, and perceived behavioral control also significantly and positively affect professional grain farmers’ intentions to grow high-quality rice, as suggested by Ajzen’s TPB (32). According to the size of path coefficients, perceived behavior control has the most significant influence, followed by perceived usefulness, which is much more important than any other variable. This contrasts with Li et al.’s perspective, which suggests that behavioral attitudes are the most critical factor in farmers’ willingness and behavior to adopt agricultural technologies, with perceived behavioral control having the least impact (57). These discrepancies may stem from varying situational factors and require further validation. Professional grain farmers require specific resources, such as funding and technical training, to cultivate high-quality rice. If farmers perceive these resources as readily available, they are more inclined to attempt cultivating high-quality rice. Furthermore, professional grain farmers, as rational economic agents, typically weigh the economic benefits of high-quality rice varieties when making their choices, aiming to maximize economic gains by minimizing costs.

The study also examined the mediating roles of perceived ease of use and perceived usefulness through behavioral attitudes and perceived behavioral control on the willingness to grow high-quality rice. These findings reveal that “perceived usefulness is positively affecting the intention of professional grain farmers to grow quality rice through behavior attitude, whereas perceived ease of use indirectly affects willingness through behavioral attitude and perceived behavioral control.” This insight elucidates how cognitive assessments translate into actual behavior in agricultural technology adoption decisions, consistent with Kanesh et al. (58) conclusions. In addition, the mediating effect value presents that “the perceived ease of use→perceived behavior control→willingness to grow high-quality rice” coefficient is the largest. That indicates professional grain farmers give the first priority to whether the implementation of new rice varieties is easy. If farmers think cultivating high-quality rice is easy and manageable, they will feel capable and confident in adopting new rice varieties.

Multi-group analysis reveals the differences in mechanisms that drive the willingness to grow high-quality rice by professional grain farmers with different levels of endowment. For high-endowment professional grain farmers, their behavioral attitudes and perceived behavioral control play more influential roles in stimulating the willingness to grow high-quality rice than for low-endowment farmers. Farmers with larger endowments and higher levels of educational attainment have higher abilities to understand and capture the benefits of high-quality rice production, which includes enhanced economic returns and higher yields of rice. Moreover, higher-income professional grain farmers can better invest and deploy technology for the better management of high-quality rice production, thus enhancing success rates and profitability from this activity and reducing its uncertainty risks. By comparison, low-endowment professional grain farmers, due to their limited knowledge of new rice variety technologies and lower risk tolerance, exhibit lower perceived behavioral control and a more cautious attitude toward new rice varieties. Consequently, the willingness of high-endowment professional grain farmers to cultivate high-quality rice is more strongly influenced by behavioral attitudes and perceived behavioral control. In the case of resource-poor professional grain farmers, subjective norm and perceived ease of use play a greater role. This can be attributed to the fact that they tend not to rely as much on independent decisions as on the opinions and behaviors of family members and peers within their communities (59). In rural communities, the social climate and the demonstration effect play a pivotal role in shaping farmers’ perceptions and decision-making processes (60). A positive social environment that embraces technological adoption can significantly enhance farmers’ receptiveness to the advantages offered by such technologies. In addition, low-endowment professional grain farmers usually perceive the ease of use of high-quality rice varieties to be slightly higher than that of high-endowment farmers. The reason may be that the low-endowment farmers usually have smaller cultivation areas and thus lower relative costs when adopting new rice varieties, leading to their higher intention to plant high-quality rice.

## 6 Conclusion and policy implications

This research utilized an integrated framework combining the Technology Acceptance Model (TAM) and the Theory of Planned Behavior (TPB) to thoroughly examine the willingness to cultivate high-quality rice among 660 professional grain farmers in Jiangxi Province, China. The results illustrate how the subjective psychological factors of these farmers affect their willingness to engage in high-quality rice cultivation and the underlying mechanisms, offering valuable insights for promoting the adoption of high-quality rice varieties. Specifically, factors such as perceived ease of use, perceived usefulness, behavioral attitude, subjective norms, and perceived behavioral control all significantly and positively influence the willingness of professional grain farmers to cultivate high-quality rice. Notably, perceived behavioral control exerts the most substantial impact, indicating that farmers’ perceptions of their ability to manage the necessary resources and technologies are critical for successful high-quality rice cultivation. Following this, perceived usefulness reflects that farmers’ recognition of the tangible benefits associated with high-quality rice cultivation also plays a significant role in shaping their willingness to pursue this practice. Mediation analysis reveals that perceived usefulness positively affects the willingness to cultivate high-quality rice through behavioral attitude, while perceived ease of use indirectly influences this willingness via both behavioral attitude and perceived behavioral control. The mediation pathway “perceived ease of use → perceived behavioral control → willingness to cultivate high-quality rice” demonstrates the strongest effect, underscoring the importance that professional grain farmers place on the ease of use and controllability of the cultivation process when deciding to grow high-quality rice. Furthermore, multi-group analysis indicates that the effects of behavioral attitude and perceived behavioral control on the willingness to cultivate high-quality rice are more pronounced among high-ability professional grain farmers. This may be due to their higher educational attainment and better access to resources, which enable them to comprehend and master high-quality rice cultivation techniques more effectively, leading to a more proactive adoption of these practices. In contrast, for low-ability professional grain farmers, subjective norms and perceived ease of use exert a more significant influence on their willingness to cultivate high-quality rice, suggesting that they are more affected by the opinions of those around them and are more sensitive to the perceived challenges of the cultivation process.

Based on these results, several recommendations are proposed:

1. Research institutions and farmers must foster stronger collaboration. Research institutions should prioritize the enhancement of training and education on new rice varieties, empowering farmers to fully comprehend and appreciate the practical value of high-quality rice. This approach will heighten their awareness of the technical, economic, social, and ecological advantages, thereby improving their perceived usefulness and attitudes toward new rice varieties.
2. Agricultural technology extension departments should establish robust communication networks with farmers. By leveraging the influence of extension personnel, farmers can be promptly informed about cultivation techniques, reducing perceived learning difficulty and enhancing perceived behavioral control. Additionally, a platform for farmers’ production exchanges should be created to foster cooperation, promote high-quality rice varieties, and encourage participation in cooperatives, thereby improving perceived ease of use and boosting confidence in technology mastery.
3. The government should tailor policies to the varying endowments of farmers. Regular assessments of attitudes and perceptions toward new rice varieties among different groups should inform timely policy adjustments, ensuring more accurate targeting and promoting the adoption of high-quality rice varieties.

This study presents a novel theoretical framework and empirical findings regarding the willingness to cultivate high-quality rice; however, it has certain limitations. Firstly, the research employed an integrated model that combines the Technology Acceptance Model (TAM) and the Theory of Planned Behavior (TPB). While this model sheds light on the factors influencing professional grain farmers’ willingness to grow high-quality rice, it does not utilize the more comprehensive C-TAM-TPB model. The C-TAM-TPB model, which integrates TAM and TPB, places a greater emphasis on the impact of external variables on individual behavioral intentions. Future research could adopt the C-TAM-TPB model to more thoroughly investigate the mechanisms driving professional grain farmers’ willingness to cultivate high-quality rice, thereby enhancing the model’s explanatory and predictive capabilities. Secondly, the sample for this study is limited to professional grain farmers in Jiangxi Province. Although Jiangxi is a significant grain-producing area in China and possesses a degree of representativeness, a sample limited to a single province may not adequately capture the variations in willingness to cultivate high-quality rice among professional grain farmers across different countries and regions. This limitation may result in the findings not being fully representative of other contexts. To enhance the generalizability and scientific robustness of the conclusions, it is essential to expand the sample size. Consequently, future efforts will focus on broadening the survey area and increasing the sample size. Lastly, the structural equation model utilized in this study primarily focuses on the core variables of the Technology Acceptance Model (TAM) and the Theory of Planned Behavior (TPB), excluding control variables. While this approach clarifies the relationships among the main factors, the absence of control variables may result in an incomplete evaluation of the factors influencing professional grain farmers’ willingness to cultivate high-quality rice. Future research should consider incorporating relevant control variables when developing structural equation models to provide a more comprehensive assessment of the various factors affecting professional grain farmers’ willingness to cultivate high-quality rice, thereby further enhancing the model’s explanatory power.

## Data Availability

The raw data supporting the conclusions of this article will be made available by the authors, without undue reservation.
