# Supplementary material for: Research on farmers’ willingness to grow high-quality rice based on the TAM-TPB model: evidence from China
Source: Front Nutr. 2025 Jul 2;12:1535720. doi: 10.3389/fnut.2025.1535720 (PMC12263639; doi:10.3389/fnut.2025.1535720)
Supplement: Supplementary file 1 [file Data_Sheet_1.DOC]

Supplementary Material

# 1 Supplementary Figures and Tables

## Supplementary Figures


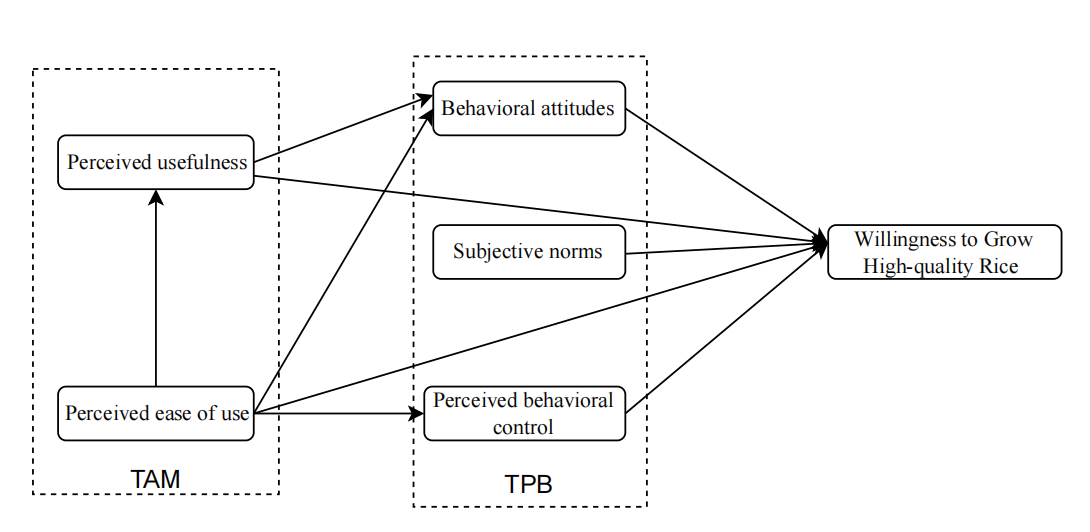
**Figure 1.** Research framework


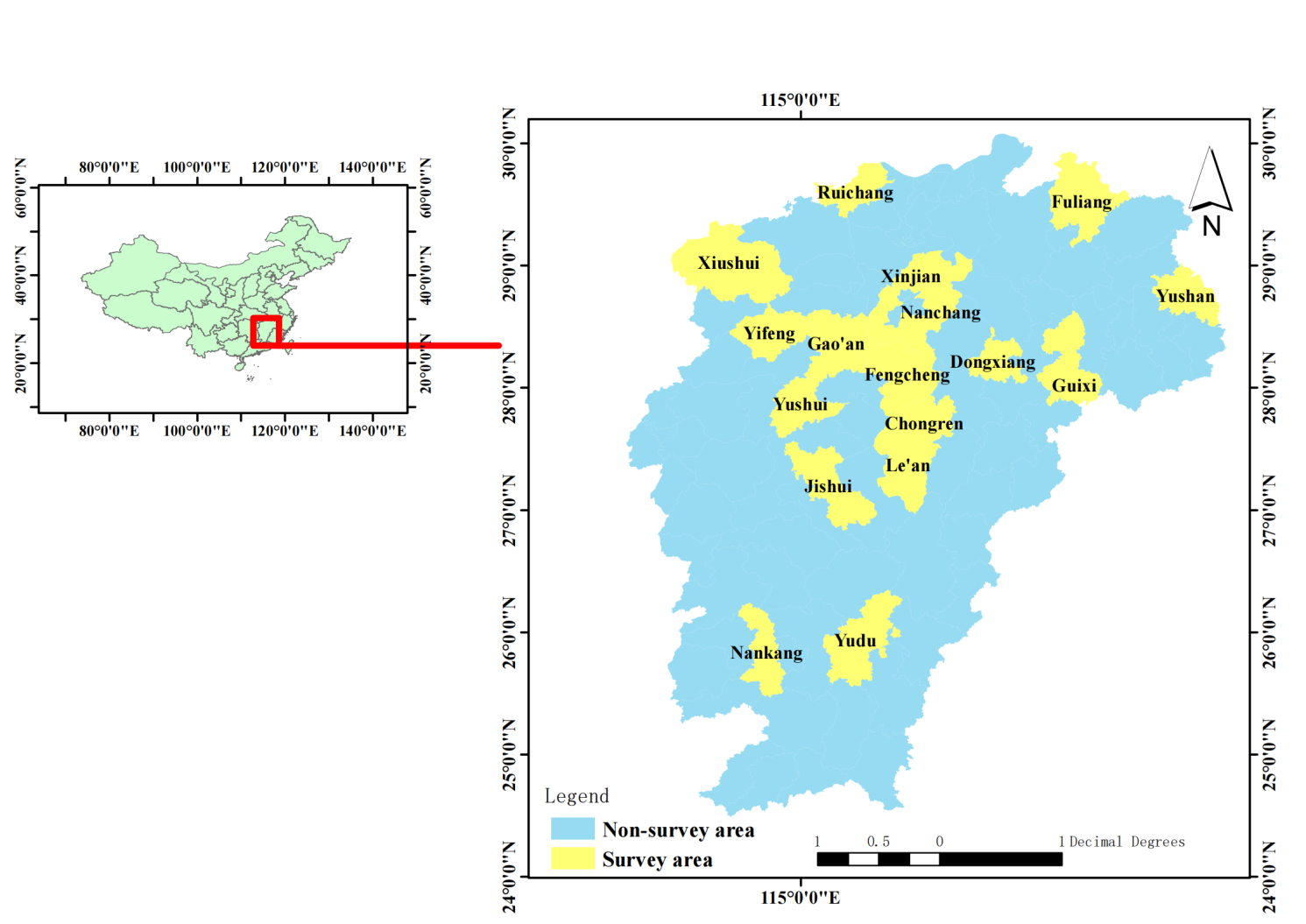


**Figure 2.** Research area


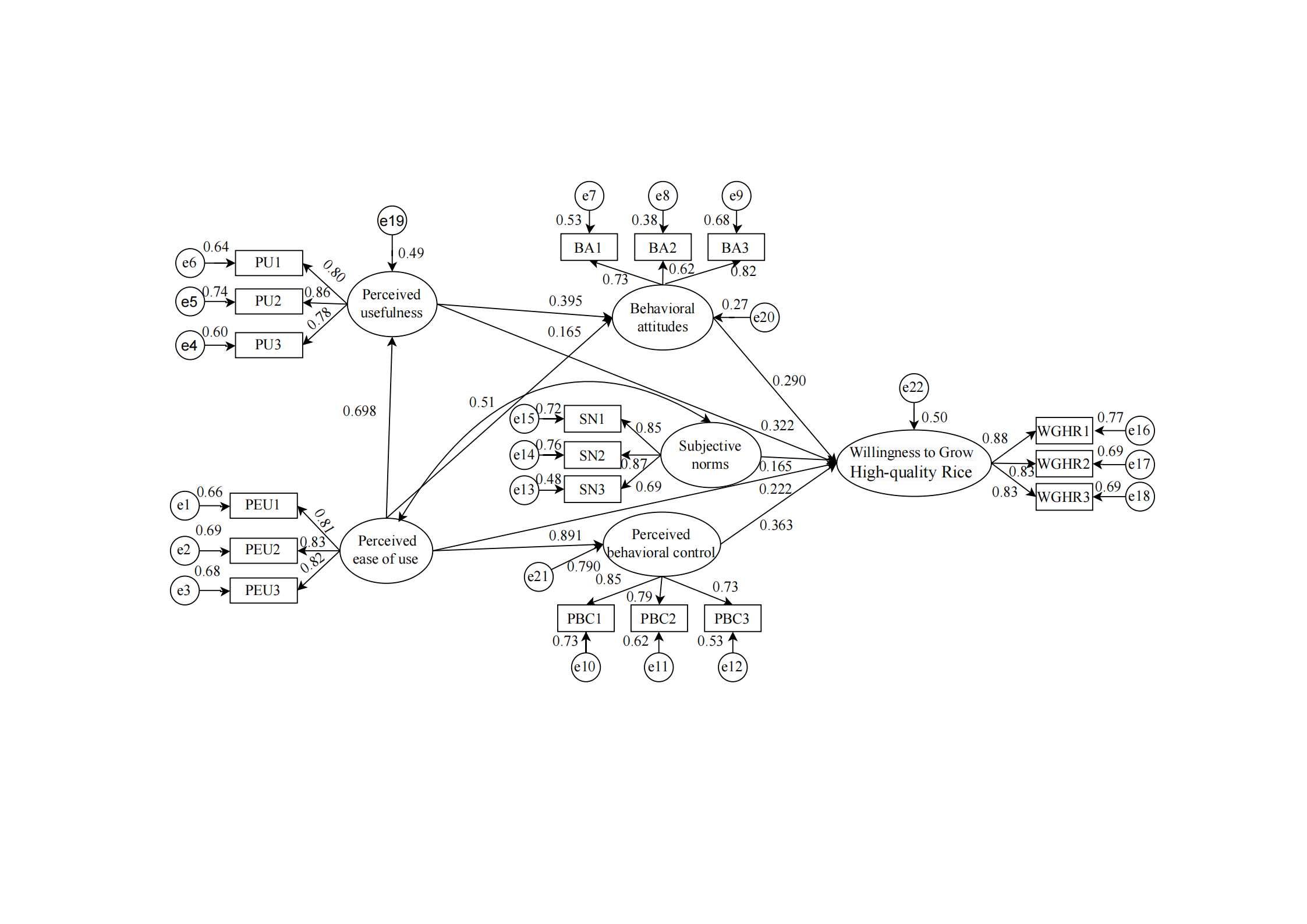
**Figure 3.** Structural Equation Model of willingness to grow high-quality rice

## Supplementary Tables

**Table 1.** Questionnaire

| **Constructs** |  | **Measurement items** | **References** |
| --- | --- | --- | --- |
| Perceived Ease of Use (PEU) | PEU1 | You believe that growing high-quality rice is relatively straightforward. | Pavlou (2003)  Chen et al. (2024b) |
|  | PEU2 | Growing high-quality rice does not require excessive effort. |  |
|  | PEU3 | You can easily obtain human resources and rice cultivation techniques. |  |
| Perceived Usefulness (PU) | PU1 | Growing high-quality rice can increase household income. | Pavlou (2003)  Chen et al. (2024b) |
|  | PU2 | Growing high-quality rice can improve the family's living environment. |  |
|  | PU3 | Growing high-quality rice can enhance rice yield. |  |
| Behavioral Attitudes (BA) | BA1 | There is a psychological expectation of profit from high-quality rice. | Xia et al. (2023)  Senger et al. (2017)  Tama et al. (2021) |
|  | BA2 | Growing high-quality rice can improve market competitiveness. |  |
|  | BA3 | You believe that growing high-quality rice is highly effective in improving rice quality. |  |
| Perceived Behavirol Control  (PBC) | PBC1 | You feel capable of growing high-quality rice. | Xia et al. (2023)  Senger et al. (2017)  Tama et al. (2021) |
|  | PBC2 | You can obtain sufficient resources to grow high-quality rice (such as funds, knowledge, etc.). |  |
|  | PBC3 | Under your conditions, successfully growing high-quality rice is not difficult. |  |
| Subjective Norms (SN) | SN1 | Your family believes you should grow high-quality rice. | Xia et al. (2023)  Senger et al. (2017)  Tama et al. (2021) |
|  | SN2 | Your fellow farmers generally support growing high-quality rice. |  |
|  | SN3 | Agricultural experts or advisors recommend that farmers grow high-quality rice to increase income. |  |
| Willingness to Grow High-quality Rice (WGHR) | WGHR1 | You are able to actively participate in growing high-quality rice. | Peng et al. (2022) |
|  | WGHR2 | You can systematically learn high-quality rice cultivation techniques. |  |
|  | WGHR3 | You will grow high-quality rice under the guidance of professionals. |  |

**Table 2. Reliability and validity analysis**

| **Constructs** | **Items** | **S.Estimate** | **Estimate** | **S.E.** | **C.R.** | **P** | **SMC** | **CR** | **AVE** |
| --- | --- | --- | --- | --- | --- | --- | --- | --- | --- |
| Perceived Ease of Use | PEU1 | 0.823 | 1 |  |  |  | 0.677 | 0.861 | 0.675 |
|  | PEU2 | 0.829 | 0.997 | 0.04 | 25.09 | *** | 0.687 |  |  |
|  | PEU3 | 0.812 | 1.092 | 0.048 | 22.739 | *** | 0.659 |  |  |
| Perceived Usefulness | PU1 | 0.776 | 1 |  |  |  | 0.602 | 0.854 | 0.661 |
|  | PU2 | 0.859 | 0.962 | 0.043 | 22.139 | *** | 0.738 |  |  |
|  | PEU3 | 0.812 | 1.092 | 0.048 | 22.739 | *** | 0.659 |  |  |
| Behavioral Attitudes (BA) | BA1 | 0.729 | 1 |  |  |  | 0.531 | 0.769 | 0.530 |
|  | BA2 | 0.616 | 0.895 | 0.07 | 12.762 | *** | 0.379 |  |  |
|  | BA3 | 0.824 | 1.214 | 0.082 | 14.762 | *** | 0.679 |  |  |
| Perceived Behavirol Control  (PBC) | PBC1 | 0.728 | 1 |  |  |  | 0.530 | 0.833 | 0.626 |
|  | PBC2 | 0.787 | 1.161 | 0.061 | 18.978 | *** | 0.619 |  |  |
|  | PBC3 | 0.853 | 1.147 | 0.058 | 19.74 | *** | 0.728 |  |  |
| Subjective Norms (SN) | SN1 | 0.694 | 1 |  |  |  | 0.482 | 0.850 | 0.656 |
|  | SN2 | 0.874 | 1.156 | 0.061 | 18.919 | *** | 0.764 |  |  |
|  | SN3 | 0.85 | 1.184 | 0.063 | 18.787 | *** | 0.723 |  |  |
| Willingness to Grow High-quality Rice (WGHR) | WGHR1 | 0.878 | 1 |  |  |  | 0.771 | 0.883 | 0.716 |
|  | WGHR2 | 0.829 | 0.837 | 0.032 | 26.333 | *** | 0.687 |  |  |
|  | WGHR3 | 0.83 | 0.938 | 0.035 | 26.485 | *** | 0.689 |  |  |

Table 3. Discriminant validity

|  | **AVE** | **PEU** | **PU** | **PBC** | **SN** | **BA** | **WGHR** |
| --- | --- | --- | --- | --- | --- | --- | --- |
| PEU | 0.675 | **0.822** |  |  |  |  |  |
| PU | 0.661 | 0.698*** | **0.813** |  |  |  |  |
| PBC | 0.626 | 0.732*** | 0.622 | **0.791** |  |  |  |
| SN | 0.656 | 0.512 | 0.357 | 0.456 | **0.810** |  |  |
| BA | 0.53 | 0.442** | 0.511*** | 0.393 | 0.226 | **0.728** |  |
| WGHR | 0.716 | 0.539* | 0.6*** | 0.555** | 0.398*** | 0.53*** | **0.846** |

Note： *, ** and *** indicate the significate level of 10%, 5% and 1%, respectively. Values on diagonal indicate the square root of the AVE.

**Table 4. Common method variance test results**

| **Model** | **CMIN** | **DF** | **CMIN/DF** | **RMSEA** | **NFI** | **GFI** | **CFI** |
| --- | --- | --- | --- | --- | --- | --- | --- |
| Six-Factor Model  PEU，PU，BA，PBC，SN，WGHR | 481.961 | 120 | 4.002 | 0.068 | 0.932 | 0.924 | 0.948 |
| Five-Factor Model  PEU，PU，BA，PBC，SN+WGHR | 1150.952 | 125 | 9.208 | 0.112 | 0.838 | 0.822 | 0.852 |
| Four-Factor Model  PEU,PU,BA,PBC+SN+WGHR | 1749.144 | 129 | 13.559 | 0.138 | 0.754 | 0.737 | 0.767 |
| Three-Factor Model  PEU,PU,BA+PBC+SN+WGHR | 2058.146 | 132 | 15.592 | 0.149 | 0.710 | 0.708 | 0.723 |
| Two-Factor Model  PEU,PU+BA+PBC+SN+WGHR | 2329.942 | 134 | 17.388 | 0.158 | 0.672 | 0.675 | 0.684 |
| Single-Factor Model  PEU+PU+BA+PBC+SN+WGHR | 2615.337 | 135 | 19.373 | 0.167 | 0.632 | 0.652 | 0.643 |

Note：Model adaptation indicators：CMIN/DF<5,RMSEA<0.08,NFI≥0.8,GFI≥0.8,CFI>0.9.

Table 5. Results of the structural model analysis

| **Relationship** | **S.Estimate** | **Estimate** | **S.E.** | **C.R.** | **Sig.** | **Result** |
| --- | --- | --- | --- | --- | --- | --- |
| PEU→PU | 0.698 | 0.868 | 0.058 | 15.026 | *** | Supported |
| PU→BA | 0.395 | 0.415 | 0.075 | 5.51 | *** | Supported |
| PEU→PBC | 0.891 | 0.87 | 0.051 | 17.163 | *** | Supported |
| PEU→BA | 0.165 | 0.216 | 0.085 | 2.534 | ** | Supported |
| BA→WGHR | 0.29 | 0.332 | 0.053 | 6.204 | *** | Supported |
| SN→WGHR | 0.165 | 0.222 | 0.058 | 3.858 | *** | Supported |
| PBC→WGHR | 0.363 | 0.555 | 0.175 | 3.166 | ** | Supported |
| PEU→WGHR | 0.222 | 0.332 | 0.187 | 1.771 | * | Supported |
| PEU→WGHR | 0.322 | 0.387 | 0.072 | 5.339 | *** | Supported |

Note： *, ** and *** indicate the significate level of 10%, 5% and 1%, respectively.

Table 6. Results of the mediation effect test

| **Paths** | **Coefficient** | **Bias-corrected 90% CI** | | |
| --- | --- | --- | --- | --- |
|  |  | **Lower** | **Upper** | **P** |
| PU→BA→WGHR | 0.138 | 0.073 | 0.240 | 0.000 |
| PEU→BA→WGHR | 0.072 | 0.017 | 0.159 | 0.031 |
| PEU→PBC→WGHR | 0.483 | 0.071 | 0.956 | 0.055 |
| Total effect | 1.148 | 0.668 | 1.595 | 0.002 |

**Table 7. Hierarchical Clustering results**

| **Group** | **Sample Size** | **Years of education** | **Annual Per Capita Household Income**  **(in ten thousand yuan)** | **Paddy Field Area (hectares)** |
| --- | --- | --- | --- | --- |
| Low-Endowment | 320 | 4.68 | 3.42 | 6.52 |
| High-Endowment | 340 | 6.54 | 6.7 | 10.88 |

Table 8. Results of multi-group analysis

| **Paths** | **High-Endowment** | | **Low-Endowment** | |
| --- | --- | --- | --- | --- |
|  | **Coefficient** | **P** | **Coefficient** | **P** |
| PEU→PU | 0.704 | *** | 0.687 | *** |
| PEU→BA | 0.377 | ns | 0.417 | ns |
| PEU→PBC | 0.889 | *** | 0.886 | ** |
| PEU→BA | 0.16 | ** | 0.173 | * |
| BA→WGHR | 0.318 | *** | 0.282 | ** |
| SN→WGHR | 0.378 | *** | 0.565 | *** |
| PBC→WGHR | 0.380 | *** | 0.315 | *** |
| PEU→WGHR | 0.156 | *** | 0.221 | *** |
| PU→WGHR | 0.235 | ** | 0.224 | ** |

Note： *， ** and *** indicate the significate level of 10%， 5% and 1%， respectively; ns indicates that the P-value is not significant.
